# KLF5 and p53 comprise an incoherent feed-forward loop directing cell-fate decisions following stress

**DOI:** 10.1038/s41419-023-05731-1

**Published:** 2023-05-02

**Authors:** Yizeng Yang, Dharmendra Bhargava, Xiao Chen, Taicheng Zhou, Gizem Dursuk, Wenpeng Jiang, Jinshen Wang, Zhen Zong, Sharyn I. Katz, Gwen A. Lomberk, Raul A. Urrutia, Jonathan P. Katz

**Affiliations:** 1 Division of Gastroenterology, University of Pennsylvania Perelman School of Medicine, Philadelphia, PA 19104 USA; 2 Department of Radiology, University of Pennsylvania Perelman School of Medicine, Philadelphia, PA 19104 USA; 3 Department of Surgery, Medical College of Wisconsin, Milwaukee, WI 53226 USA; 4 Genomic Sciences and Precision Medicine Center, Medical College of Wisconsin, Milwaukee, WI 53226 USA

**Keywords:** Apoptosis, Stress signalling

## Abstract

In response to stress, cells make a critical decision to arrest or undergo apoptosis, mediated in large part by the tumor suppressor p53. Yet the mechanisms of these cell fate decisions remain largely unknown, particularly in normal cells. Here, we define an incoherent feed-forward loop in non-transformed human squamous epithelial cells involving p53 and the zinc-finger transcription factor KLF5 that dictates responses to differing levels of cellular stress from UV irradiation or oxidative stress. In normal unstressed human squamous epithelial cells, KLF5 complexes with SIN3A and HDAC2 repress *TP53*, allowing cells to proliferate. With moderate stress, this complex is disrupted, and *TP53* is induced; KLF5 then acts as a molecular switch for p53 function by transactivating AKT1 and AKT3, which direct cells toward survival. By contrast, severe stress results in KLF5 loss, such that AKT1 and AKT3 are not induced, and cells preferentially undergo apoptosis. Thus, in human squamous epithelial cells, KLF5 gates the response to UV or oxidative stress to determine the p53 output of growth arrest or apoptosis.

## Introduction

Epithelial cells are continuously challenged by genotoxic stresses and are the origins of most human cancers [1, 2]. DNA damage caused by these stresses induces several cellular responses, including growth arrest to prevent the replication of damaged DNA, and apoptosis, which eliminates aberrant cells [3, 4]. The decision of each cell to arrest or undergo apoptosis is determined, to a great extent, by the level of the insult [5], but the specific mechanism by which the cell makes this decision is not clear. Importantly, the malignant transformation of a single epithelial cell is a rare event, indicating that the regulation of the DNA damage response and the processes preventing normal epithelial cells from becoming cancer cells are exquisitely controlled [6]. The tumor suppressor p53, the “guardian of the genome,” is an important mediator of the DNA damage response and protects against malignant transformation in normal human epithelia [7, 8]. In normal epithelial cells that are actively proliferating, p53 levels are low, allowing these cells to progress through the cell cycle [9]. When cells are stressed, p53 is induced, and, once induced, its function is regulated by a number of mechanisms, including the E3 ubiquitin ligases MDM2 and MDMX [4, 7, 10]. Yet the factors that regulate *TP53* expression at the transcriptional level and the mechanisms of p53-mediated cell-fate decisions in response to stress remain largely unknown [4, 11, 12].

p53 is the most commonly mutated gene in human cancers, and p53 function and dysfunction have been extensively studied in cancer cells [8]. In normal epithelial cells, understanding the mechanisms of p53-mediated cell-fate decisions in response to stress would have a profound impact on chemoprevention and potentially define new targets for therapy [12]. Some have speculated that p53 levels are the key determinants of cellular output, with low levels of p53 in response to “everyday exposure” to stresses favoring cell cycle arrest and damage repair, while more severe stress leads to irreparable damage and apoptosis [4]. In this model, the downstream consequences of p53 induction are the activation of specific target genes that drive the cells toward either growth arrest or apoptosis [12]. But how does p53 select which genes to activate in these contexts [11]? Clearly, tight regulation of the DNA damage response is essential to ensure, for example, that damaged cells that cannot be repaired are not allowed to survive and potentially proliferate.

Clues to the mechanisms by which p53 mediates these critical cell-fate decisions might come from studies of other key transcriptional regulators of the DNA damage response that interact with p53. The zinc-finger transcription factor KLF5 is an important regulator of cell cycle progression and apoptosis that interacts with p53 in multiple contexts [13–16]. In normal epithelial cells, KLF5 promotes proliferation, and KLF5 is a key mediator of the stress response in normal tissues [17, 18]. In epithelial tumorigenesis, KLF5 functions may vary, including by tissue or tumor type, and p53 may be key for these context-dependent functions [16, 17]. For example, KLF5 and p53 coordinately regulate *NOTCH1* to suppress malignant transformation in normal squamous epithelial cells [13], and KLF5 and p53 also functionally interact in cancer cells, regulating *HIF1α* expression in colon cancer [15] and *survivin* expression in acute lymphoblastic leukemia [14]. In addition, mutant p53 alters KLF5 functions in both cellular proliferation and malignant transformation [13, 19]. Thus, interactions of KLF5 and p53 are critical in both normal and cancer cells. However, while p53, both wild-type and mutant, can regulate KLF5 function, whether KLF5 controls p53 expression and/or function in normal cells, during proliferation, and in response to stress is not known. Moreover, significant questions remain about the “network architecture” of p53 in response to stress [20].

Here, using non-transformed primary human squamous epithelial cells, we define an incoherent feed-forward loop involving p53 and KLF5 that gates cellular responses to different levels of stress. We show that in normal unstressed cells, KLF5 forms a repressive complex on *TP53*, allowing cells to proliferate unchecked by the growth-inhibitory effects of p53 [4, 9]. In response to moderate stress, this complex is disrupted, and p53 is induced; p53 is also induced in severe stress but in association with the loss of KLF5. In cells under stress, KLF5 acts as a molecular switch for p53 function: when KLF5 is present, both KLF5 and p53 act together to transactivate AKT1 and AKT3, which directs cells toward survival rather than apoptosis; when KLF5 is absent, AKT1 and AKT3 are not induced, and cells preferentially undergo apoptosis. Thus, we demonstrate that KLF5 is critical for *TP53* repression during normal cell proliferation and for determining the p53 output of growth arrest or apoptosis in response to cellular stress.

## Materials and methods

### Cell culture

Human esophageal squamous epithelial cells (keratinocytes) were a gift of Dr. Anil Rustgi (Columbia University), human cervical keratinocytes were a gift from Dr. Craig Meyers (Penn State College of Medicine), and human skin keratinocytes were purchased from ATCC. Primary keratinocytes were grown at 37 °C and 5% CO_2_ in keratinocyte serum-free medium (K-SFM; ThermoFisher Scientific), supplemented with 40 μg/mL bovine pituitary extract (Invitrogen), 1.0 ng/mL epidermal growth factor (EGF; Invitrogen), 100 U/mL penicillin, and 100 μg/mL streptomycin (Invitrogen). The medium was changed every 24 h for 5 days. For UV experiments, cells were treated in a Spectroline UV crosslinker without filtering. Unless noted, cells were harvested 8 h after UV irradiation or hydrogen peroxide treatment. For treatment with trichostatin A (Cayman Chemical), cells were harvested after 16 h.

### shRNA viral constructs and transduction

The Tet-On Lentiviral vector pTripz (Dharmacon) was used initially to express distinct short hairpin RNAs (shRNA) against *KLF5* (Dharmacon; catalog No. RHS4740). Additional shRNA was generated by cloning into EZ-Tet-pLKO-Blast (gift from Cindy Miranti, Addgene plasmid #85973). shRNA against *SIN3A* in the pKLO lentiviral vector was purchased from Sigma-Aldrich. Lentivirus has been packaged in HEK 293 T cells with the lentiviral packaging plasmids pCMV-dR8.74 and pMD2.G (gift from Dr. Didier Trono, Addgene plasmid #12259). Depending upon the vector utilized, non-silencing inducible shRNA in TRIPZ (Dharmacon; catalog No. RHS4743) or EZ-Tet-pLKO-Blast was used as a control for shRNA experiments. Infected cells were selected with 1 µg/ml puromycin for 14 days or 20 µg/ml blasticidin for 21 days and induced with 2 µg/ml doxycycline. Except as stated, experiments were performed after 48 h of doxycycline induction. shRNA sequences are listed in Table S1.

### siRNA transfection

Cells were grown as a monolayer on a 6-well plate and transfected the following day with *HDAC2*, *TP53*, or negative control siRNA (Table S2) using PepMute™ siRNA Transfection Reagent (Signagen). Cells were harvested for analyses 72 h after transfection.

### Quantitative real-time PCR

Total RNA was isolated with the RNeasy micro kit (Qiagen), and cDNA was synthesized with the High-Capacity cDNA Reverse Transcription Kit (Life Technologies). Quantitative real-time PCR was performed in triplicate on three samples for each experimental condition on a StepOne Plus Real-Time PCR System (Life Technologies) using Power SYBR Green Master Mix (Life Technologies). TATA-box-binding protein (TBP) served as an internal control. Primer sequences are listed in Table S2.

### Western blotting and mass spectrometry

Totally, 30 µg of total protein was separated for each sample on a Nupage 4–12% Bis–Tris acrylamide gel (ThermoFisher Scientific) and transferred onto a polyvinylidene difluoride membrane (Millipore). Membranes were probed with 1:5,000 rabbit anti-KLF5 antibodies [21] or 1:5000 rabbit anti-KLF5 antibodies (#21017-1-AP, Proteintech), 1:2,000 mouse anti-p53 antibody (#48818S, Cell Signaling Technology), 1:1000 rabbit anti-BCL2 (#2870, Cell Signaling Technology), 1:1000 rabbit anti-BAX (#2772, Cell Signaling Technology), 1:2000 rabbit anti-SIN3A (#7691S, Cell Signaling Technology), 1:2000 mouse anti-HDAC2 antibody (#5113S, Cell Signaling Technology), 1:3000 anti-rabbit AKT (#9272, Cell Signaling Technology), or 1:1000 anti-rabbit ubiquitin (#3933S, Cell Signaling Technology) in TBST with 5% non-fat milk followed by secondary anti-rabbit or anti-mouse antibodies conjugated with horseradish peroxidase (GE Healthcare Life Sciences) and developed with Immobilon Western Chemiluminescent HRP Substrate (Millipore). Mouse anti-β-actin at 1:1000 (#3700S, Cell Signaling Technology) served as a loading control. Acetylation-specific anti-KLF5 was a gift from Dr. Jin-Tang Dong at Emory University [22]. For mass spectrometry, protein bands were excised from Coomassie blue-stained gels and submitted for gas chromatography–mass spectrometry performed by the University of Pennsylvania Proteomics and Systems Biology Core. Full and uncropped Western blots are provided in Supplementary Information.

### Co-immunoprecipitation

EPC2 cells containing shRNA against KLF5 (shKLF5-pTRIPZ-puro) or 293FT cells (Thermo Fisher) containing KLF5-WT or mutant KLF5-K369R expression plasmid [22] were seeded onto 100 mm plates. After 5 days of treatment with doxycycline, cells were harvested in lysis buffer containing 20 mM Tris-Cl ph-8, 137 mM NaCl, 2 mM EDTA,1% NP 40, a protease inhibitor (Protease Inhibitor Cocktail, Millipore Sigma), and phosphatase inhibitor (PhosSTOP, Millipore Sigma). The lysate was precleared with A/G magnetic beads (Cell Signaling Technology) for 2 h with rotation in 4 °C. Protein concentration was measured using BioRad DC protein assay. Equal amounts of lysate were taken, and 4 µg antibodies were added. The lysate was incubated with antibody overnight with rotation at 4 °C. 20 µl protein A/G magnetic beads were added to each tube and incubated for 4 h at 4 °C with rotation. The complex was then washed three times with wash buffer (10 mM tris ph-7.5, 1 mM EDTA,1 mM EGTA, 150 mM NaCl, 15 Triton X-100, 0.2 mM sodium orthovanadate, and Protease Inhibitor Cocktail) and resuspended in 1x protein loading dye and heated at 95 °C for 5 min. Five microlitres of the suspension were loaded onto the gel for immunoblotting.

### Cell viability assay

EPC2 cells expressing shRNA against KLF5 (shKLF5-pTRIPZ-puro) were seeded in 25 mm tissue culture dishes and incubated in the presence or absence of 1 µg/ml doxycycline for three days. Cells were exposed to 60 mj/cm^2^ UV in a UV crosslinker and incubated for different time points from 0 to 16 h. Cells were detached with 0.05% trypsin and collected in a 15 ml tube containing 0.05% soybean trypsin inhibitor. Cells were centrifuged and washed with PBS and resuspended in a 2 ml medium. 50 µl of 0.4% trypan blue was added to 50 µl of cells in suspension in a 1.5 ml tube. 10 µl of the cell/trypan blue suspension was loaded onto cell counting chamber slides. The slides were read on a Countess Automated Cell Counter (Thermo Fisher).

### Flow cytometry

Cells grown in a monolayer were dissociated with 0.05% trypsin (Life Technologies) and neutralized with soybean trypsin inhibitor (Sigma-Aldrich). Cells were stained with 50 µg/ml propidium iodide (Life Technologies) or 4′,6-diamidino-2-phenylindole (DAPI) in PBST buffer for cell cycle or PE annexin V-7AAD Apoptosis detection kit (Biolegend) and analyzed with a BD Biosciences Accuri C6 flow cytometer or BD LSRFortessa Cell analyzer. All assays were performed in triplicate.

### ChIP and reChIP assays

ChIP assays were performed with the ChIP assay kit (Millipore). In brief, cells were cross-linked with 1% formaldehyde for 10 min at room temperature, lysed with SDS buffer, and DNA was sheared by sonication. Samples were diluted and pre-cleared with protein A-agarose/salmon sperm for 30 min at 4 °C. DNA-protein complexes were incubated with 1:5,000 rabbit anti-KLF5 antibody [21] at 4 °C overnight and precipitated with protein A-agarose for 1 h. DNA was purified with the Qiaquick PCR purification kit (Qiagen), and quantitative PCR on the *TP53* promoter using specific primers (Fig. S1) was performed with Power SYBR Green Master Mix (Life Technologies). For ReChIP, the first round of ChIP was performed as for ChIP using an anti-KLF5 antibody (#21017-1-AP, Proteintech) and rabbit anti-IgG (#8726 S, Cell Signaling Technology). After washing, the complex was incubated for 30 min at 37 °C in 75 μL TE/10 mM DTT, eluted, and then diluted 20x with ChIP dilution buffer. A second round of ChIP was performed overnight at 4 °C with rotation using anti-SIN3A or anti-HDAC2 antibody on KLF5 IP samples or anti-IgG antibody on IgG samples, as per the manufacturer protocol.

### Luciferase reporter assays

For *TP53* reporter assays, we cloned a fragment of the *TP53* promoter containing either 1.6 or 0.6 kb upstream of the transcriptional start site into the pGL3-Basic reporter vector (Promega). The *AKT1* reporter plasmid AKT-1678 contains a 1678 bp region from the 5′ regulatory region of *AKT1* cloned into pGL3-Basic. The plasmid containing the acetylation-deficient K369R mutant of KLF5 was a gift from Dr. Jin-Tang Dong at Emory University [23]. Reporter constructs and associated plasmids were transfected into HEK293 cells with Lipofectamine 2000 (Thermo Fisher). After 40 h, cells were lysed with cell lysis buffer, and luciferase activity was measured with the Dual-Luciferase Reporter Assay System (Promega) on a microplate luminometer (Dynex Technologies). Data were normalized to Renilla activity and expressed as relative luciferase activity.

### Statistical analyses

Results are expressed as mean ± SEM, with statistical significance of differences between experimental conditions established at 95%. Student’s *t*-test or ANOVA was performed using the Analysis ToolPak for Excel (Microsoft)/ GraphPad Prism 9 software.

## Results

### KLF5 suppresses *TP53* in normal cells

**Fig. 1:**
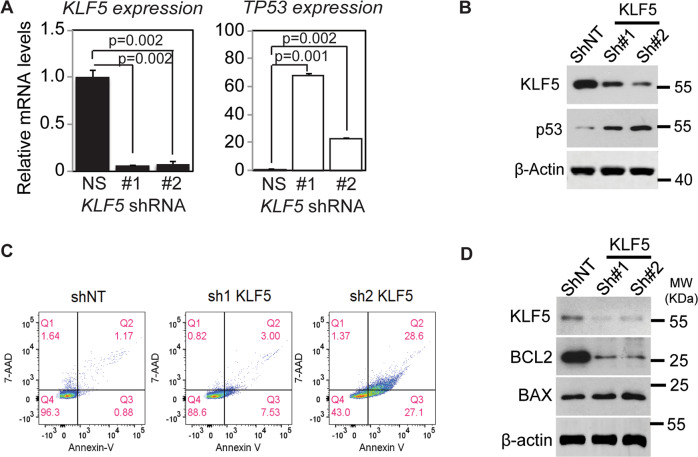
KLF5 suppresses p53 in unstressed human keratinocytes. **A** As assessed by quantitative real-time PCR, either of two shRNA directed against *KLF5* in pTRIPZ-puro increased *TP53* mRNA expression compared to non-silencing control (NS) after 7 days of doxycycline induction in primary human esophageal keratinocytes. **B** Similarly, p53 protein levels increased markedly with *KLF5* silencing with shRNA in EZ-Tet-pLKO-Blast for 7 days. β-actin served as a loading control. Western blots are representative of three independent biological experiments. **C** As demonstrated by flow cytometry with Annexin V-PE and 7-AAD, *KLF5* knockdown with shRNA in EZ-Tet-pLKO-Blast in primary human esophageal keratinocytes induced apoptosis but not necrotic cell death. **D** By western blot, antiapoptotic BCL2 was decreased significantly while proapoptotic BAX increased only slightly after 7 days of *KLF5* knockdown with shRNA in EZ-Tet-pLKO-Blast. Western blots are representative of two independent biological experiments.

KLF5 promotes cell proliferation and is normally restricted to the proliferative compartments of epithelia [18, 24], and in proliferating epithelial cells, p53 mRNA and protein are normally maintained at low levels [9]. To determine the effects of KLF5 on *TP53* in proliferating epithelial cells, we utilized inducible *KLF5* knockdown in non-transformed, primary human esophageal keratinocytes, in which p53 is wild-type and functionally intact [13, 25]. When *KLF5* was reduced following shRNA induction, p53 markedly increased, both at the mRNA and protein levels (Fig. 1A-B**)**, suggesting that in normal, proliferating cells, KLF5 represses *TP53*. In the absence of exogenous stress, *KLF5* knockdown increased apoptosis and cell cycle arrest (Fig. 1C, Fig. S2A, B). Expression of the pro-apoptotic p53 target *BAX* [4] was only slightly increased, while expression of anti-apoptotic *BCL2* was significantly reduced at the protein level (Fig. 1D); the p53 target *PUMA* [4] was also increased at the transcriptional level by *KLF5* knockdown (Fig. S2C) while other p53 targets such as *BAD*, *14-3-3*, *GADD45A*, *p21* [4] were unaffected (Fig. S2D). To determine whether p53 loss could rescue the changes in apoptosis and cell cycle induced by *KLF5* knockdown, we transfected cells containing shRNA against *KLF5* with siRNA directed against *TP53*. Loss of *TP53* in the context of *KLF5* knockdown reversed the changes seen with *KLF5* knockdown alone, decreasing apoptosis and the number of cells in G2/M (Fig. S3). Thus, KLF5 suppresses *TP53* in proliferating epithelial cells, contributing to the inhibition of apoptosis and growth arrest.

### KLF5 forms a repressive complex on the *TP53* promoter

**Fig. 2:**
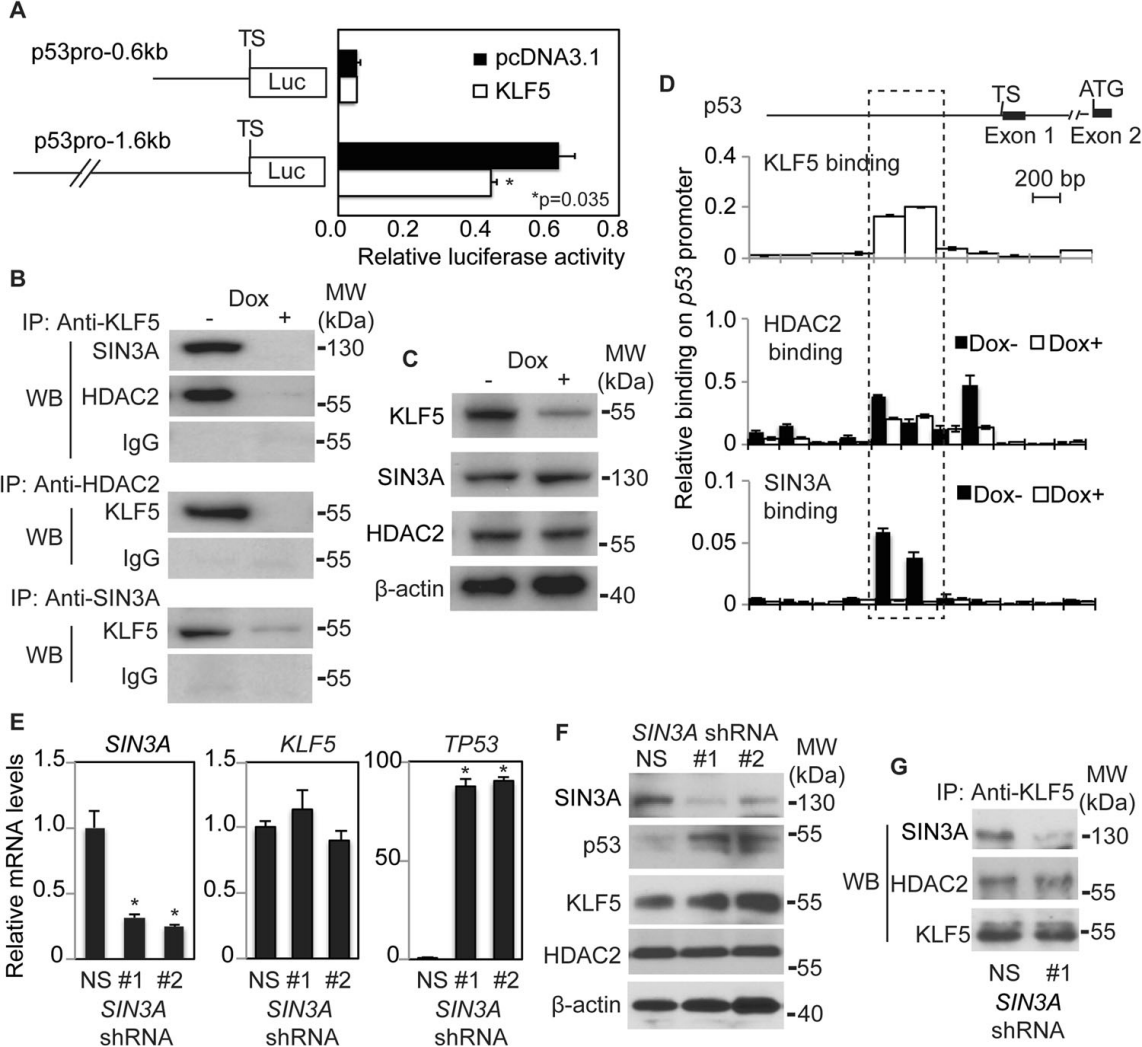
KLF5 recruits SIN3A and HDAC2 to form a repressive complex on the *TP53* promoter. **A** KLF5 inhibited the activity of a *TP53* reporter containing a 1.6 kb region upstream of the transcriptional start site. No effect of KLF5 was seen on a 0.6 kb reporter, indicating that the region from −0.6 kb to −1.6 kb was critical for KLF5 repressive functions on *TP53*. **B** KLF5 bound to SIN3A and HDAC2 in primary human esophageal keratinocytes, and this binding was inhibited by *KLF5* knockdown with shRNA in pTRIPZ-puro. IgG was used as a negative control. **C** In contrast, *KLF5* knockdown with shRNA in pTRIPZ-puro did not significantly alter SIN3A or HDAC2 levels on Western blot. β-actin was a loading control. **D** Quantitative ChIP using overlapping primers covering 4 kb of the *TP53* promoter revealed KLF5, SIN3A, and HDAC2 binding within the region from −1.4 kb to −1.0 kb. Of note, the binding of SIN3A in this region was nearly abolished with *KLF5 (shKLF5-pTRIPZ-puro)* knockdown. The binding was normalized to input DNA. **E** Compared to a non-silencing (NS) control, *SIN3A* knockdown in primary human esophageal keratinocytes with either of two shRNA constructs resulted in *TP53* induction by quantitative real-time PCR but no change in *KLF5* expression (*p < 0.001). **F** Similarly, by Western blot, *SIN3A* knockdown increased p53. Western blots are representative of two independent biological experiments. **G** While *SIN3A* knockdown abrogated KLF5-SIN3A binding, no changes were seen in KLF5-HDAC2 binding by co-immunoprecipitation.

p53 can be regulated transcriptionally and by the ubiquitin ligases MDM2 and MDMX [10, 26]. To investigate the mechanisms of p53 suppression by KLF5, we examined MDM2 and MDMX levels, as well as *TP53* mRNA stability, and found no relevant changes in any of these with *KLF5* knockdown (Fig. S4), suggesting that *KLF5* transcriptionally represses *TP53*. KLF5 did significantly reduce the activity of the *TP53* promoter, and the region between −1.6 to −0.6 kb was critical for this repression (Fig. 2A). In general, KLF5 can either activate or repress transcription; [24] other KLF family members depend upon the SIN3A/HDAC co-repressor complex for their repressive functions [27, 28]. To determine whether KLF5 physically interacts with SIN3A and/or HDAC, we performed co-immunoprecipitation with and without *KLF5* knockdown. We found that KLF5 bound to SIN3A and HDAC2 in human esophageal keratinocytes (Fig. 2B), and while KLF5 knockdown inhibited this binding, loss of KLF5 did not affect the levels of SIN3A or HDAC2 (Fig. 2C). The physical interaction of KLF5, SIN3A, and HDAC2 was also confirmed by mass spectrometry (Fig. S5A).

To demonstrate that KLF5, SIN3A, and HDAC2 complexed on the *TP53* promoter, we employed chromatin immunoprecipitation (ChIP) and quantitative PCR using a series of overlapping primers that together covered 2 kb upstream and 2 kb downstream of the transcriptional start site of *TP53*. We identified concurrent binding of KLF5, SIN3A, and HDAC on *TP53* within the region from −1.4 kb to −1.0 kb (Fig. 2D); of note, in the absence of KLF5, SIN3A binding to the 5′ regulatory region of *TP53* was lost, while HDAC2 still bound, suggesting that KLF5 is required for SIN3A binding to *TP53* while HDAC2 and KLF5 bind *TP53* independently. The binding of KLF5 with SIN3A and of KLF5 with HDAC2 on *TP53* was also demonstrated by ChIP-ReChIP (Fig. S5B). Functionally, shRNA knockdown of *SIN3A* increased the expression of p53 at both the mRNA and protein levels (Fig. 2E, F), while HDAC2 levels were unaffected. KLF5 protein levels increased slightly with *SIN3A* knockdown; as *KLF5* is not known to harbor a SIN3A binding site [29], it is not clear whether this regulation is direct. Interestingly, SIN3A was not required for the interaction between KLF5 and HDAC2 (Fig. 2G). Thus, the KLF5-SIN3A-HDAC2 complex transcriptionally represses *TP53* in unstressed primary epithelial cells.

### The repressive complex is disrupted by stress

**Fig. 3:**
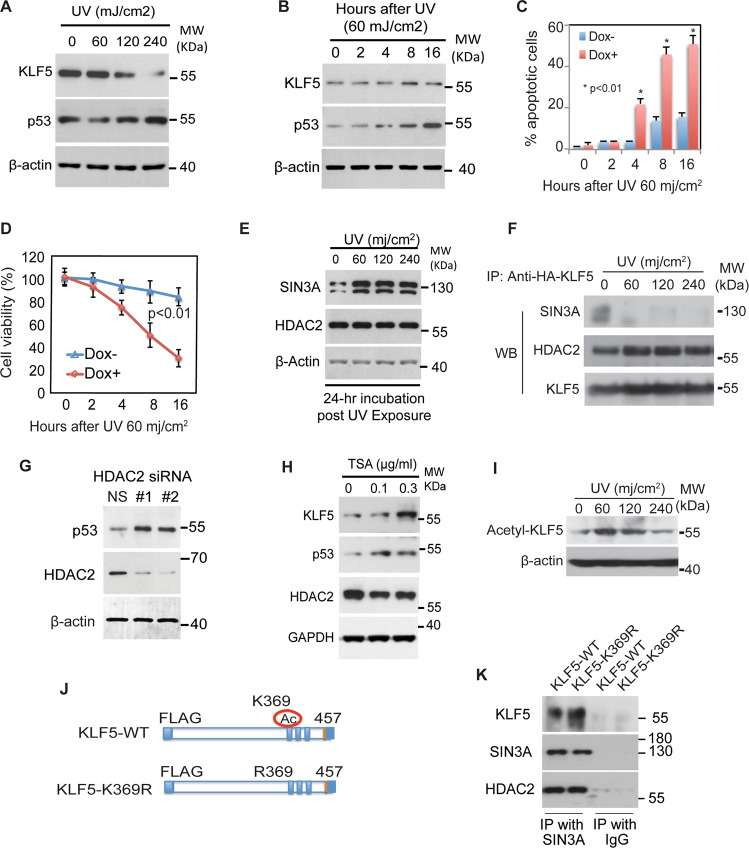
The KLF5 repressive complex on *TP53* is disrupted by stress. **A** Even in the presence of KLF5, p53 was induced by increasing UV stress in primary human esophageal keratinocytes, as shown by Western blot. β-actin served as a loading control. Western blots are representative of three independent biological experiments. **B** Both KLF5 and p53 were induced following exposure to 60 mj/cm^2^ of UV in primary human esophageal keratinocytes. β-actin served as a loading control. Western blots are representative of three independent biological experiments. **C** Apoptosis, assessed by flow cytometry with annexin V at different time points after 60 mj/cm^2^ of UV, was increased following *KLF5* knockdown with shRNA in pTRIPZ-puro by doxycycline induction. **D** *KLF5* knockdown with shRNA in pTRIPZ-puro also decreased cell viability, as assessed by trypan blue exclusion. **E** On Western blot, SIN3A levels decreased somewhat with UV stress, while HDAC2 levels were unchanged. Western blots are representative of three independent biological experiments. **F** By co-immunoprecipitation, SIN3A binding to KLF5 was abolished by UV stress, even as HDAC2 is still bound to KLF5. Western blots are representative of two independent biological experiments. **G** p53 protein levels increased on Western blot upon knockdown of *HDAC2* with siRNA. β-actin served as a loading control. Western blots are representative of two independent biological experiments. **H** p53, as well as KLF5, was induced in primary human esophageal keratinocytes treated with the HDAC inhibitor trichostatin A (TSA). Western blots are representative of three independent biological experiments. **I** KLF5 acetylation at K369 increased with stress, as assessed by Western blot with an acetylation-specific KLF5 antibody. Western blots are representative of two independent biological experiments. **J** Plasmids containing KLF5-WTor KLF5-K369R, which incorporates a lysine to arginine mutation at amino acid 369 of KLF5, were utilized for experiments. **K** As demonstrated by co-immunoprecipitation following transfection in HEK293 cells, which have low levels of endogenous KLF5, the binding of SIN3A and KLF5 was not disrupted with KLF5-K369R.

p53 and KLF5 both participate in the DNA damage response [7, 16, 18, 24]. To determine the function of KLF5 in normal cells under stress, we first exposed primary epithelial cells to ultraviolet (UV) irradiation, a model of the DNA damage response [30], and measured levels of KLF5 and p53. At lower UV doses (Fig. 3A) and early time points following UV exposure (Fig. 3B), KLF5 levels were maintained or increased, but KLF5 was subsequently lost with higher UV doses and decreased at later time points. Nonetheless, even when KLF5 was present, p53 was still induced by UV stress, indicating that, unlike in unstressed cells, KLF5 does not prevent *TP53* activation in epithelial cells under stress. Moreover, *KLF5* mRNA levels initially decreased and then increased at later time points when KLF5 protein levels were beginning to decline (Fig. S6A), suggesting that regulation of KLF5 following UV stress is posttranscriptional. In control epithelial cells, apoptosis increased coincident with p53 induction following UV treatment, and KLF5 was important for cellular responses to stress, as *KLF5* knockdown significantly increased apoptosis (Fig. 3C) and decreased cell viability (Fig. 3D). Thus, p53 expression in the absence of KLF5 is associated with increased apoptosis and decreased cell survival.

We hypothesized that *TP53* activation under stress resulted from disruption of the KLF5-SIN3A-HDAC2 repressive complex. At higher levels of stress, KLF5 was lost, and this loss of KLF5 provided an explanation for *TP53* induction at these stress levels. However, KLF5 was still present in cells exposed to low or moderate levels of stress. To determine whether the repressive complex was disrupted even in the presence of KLF5 at low to moderate stress, we examined the formation of the repressive complex by co-immunoprecipitation in cells exposed to increasing UV irradiation. SIN3A protein levels were reduced but not eliminated, and HDAC2 levels were unchanged by UV irradiation (Fig. 3E). With UV irradiation, SIN3A no longer complexed with KLF5 (Fig. 3F), providing a potential mechanism for *TP53* de-repression; this loss of SIN3A from the complex could be the result of decreased SIN3A levels, decreased binding to KLF5 or both. Interestingly, *HDAC2* knockdown (Fig. 3G) or treatment of cells with the HDAC inhibitor trichostatin A (Fig. 3H) both resulted in p53 induction, consistent with a role for HDAC in *TP53* repression; of note, this induction by trichostatin A was not mediated by KLF5 suppression with trichostatin A treatment. In addition, p53 was similarly induced in cells with oxidative stress from hydrogen peroxide, and the SIN3A repressive complex was also disrupted (Fig. S6B, C).

We hypothesized that post-translational modifications induced by stress might inhibit the assembly of the KLF5-SIN3A-HDAC2 repressive complex, adding to any effects of the reduced SIN3A levels. To determine the mechanism of complex disruption, we examined the acetylation of KLF5 at K369, which is essential for KLF5 function and degradation in other contexts [22, 24, 31, 32], using an acetylation-specific KLF5 antibody. KLF5 acetylation increased in primary epithelial cells with UV irradiation (Fig. 3I). To determine whether KLF5 acetylation was required for the formation of the repressive complex, we transfected 293FT cells, which have low endogenous KLF5 levels, with an acetylation-deficient KLF5 mutant, KLF5-K369R (Fig. 3J, Fig. S7A) [23, 24]. We found that KLF5-SIN3A binding was unaffected by K369 acetylation (Fig. 3K) and that KLF5 acetylation at K369 is not required for KLF5 repression of *TP53* (Fig. S7B). Thus, UV stress increases KLF5 acetylation at K369, and KLF5 acetylation at K369 is not required for KLF5-SIN3A binding.

### UV stress initially stabilizes KLF5

**Fig. 4:**
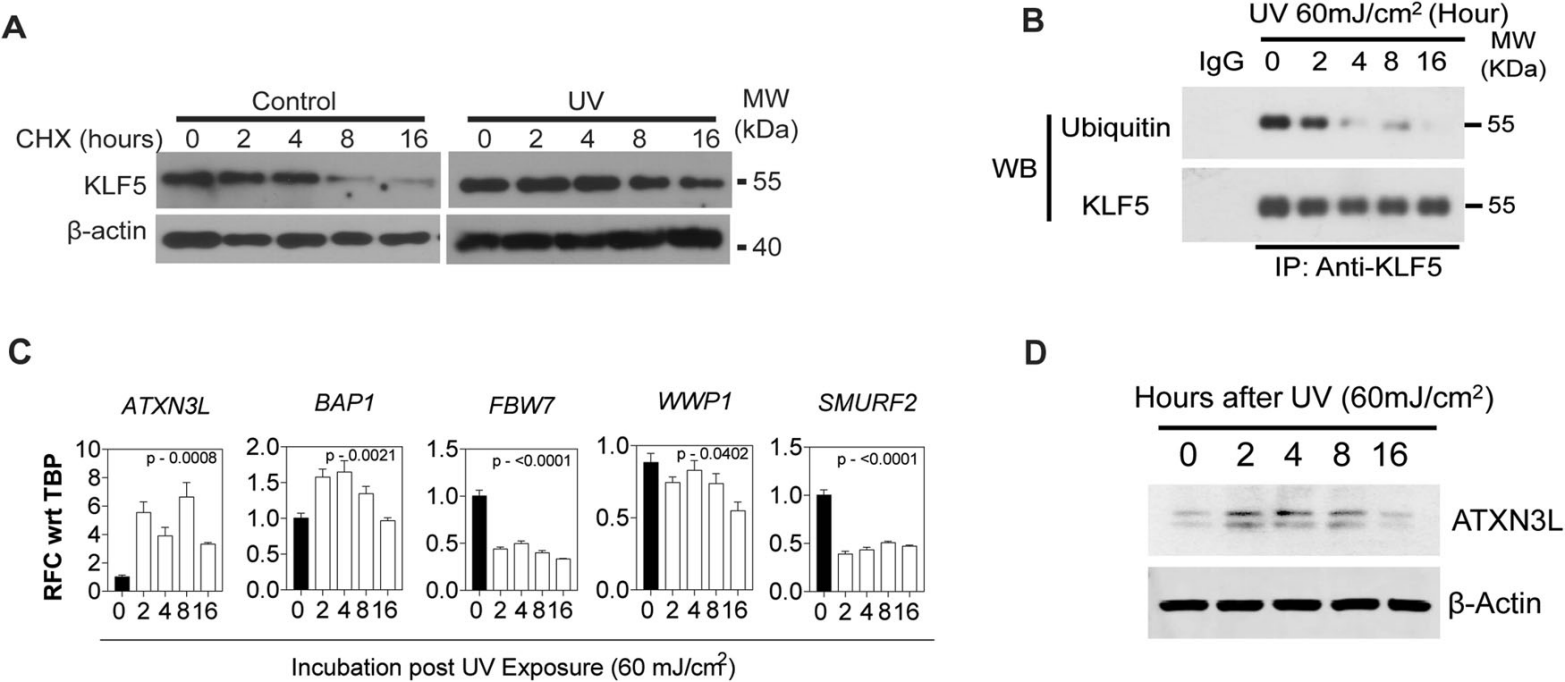
KLF5 protein is stabilized by UV stress. **A** Following UV irradiation, primary human esophageal keratinocytes were cultured with the protein synthesis-inhibitor cycloheximide (CHX) and harvested at various time points. When protein synthesis was inhibited, KLF5 decreased starting at 8 h in control cells, but this decrease was blunted in cells after UV irradiation. Western blots are representative of two independent biological experiments. **B** By co-immunoprecipitation, ubiquitination of KLF5 decreased after UV irradiation, providing a potential mechanism for KLF5 stabilization but not for KLF5 loss. Western blots are representative of three independent biological experiments. **C** By qPCR, the deubiquitinases *ATXN3L* and *BAP1* increased following UV stress while *FBW7*, *WWP1*, and *SMURF2*, ubiquitinases that regulate KLF5 levels in other contexts, were decreased. *p*-values are relative to untreated controls. **D** By Western blot, the deubiquitinase ATXN3L was also induced at the protein level following UV stress. Western blots are representative of two independent biological experiments.

Protein levels are typically controlled by a balance between protein synthesis and degradation [33]. To determine whether UV stress impacts KLF5 protein stability, we treated primary epithelial cells with cycloheximide, an inhibitor of protein biosynthesis. In control cells treated with cycloheximide, KLF5 levels decreased more than 50% by 8 h, an effect that was blunted in UV-treated cells (Fig. 4A); these findings are consistent with an important role for KLF5 in the early stress response, although the mechanism for KLF5 loss with higher levels of UV stress was not clear. In cancer cells, KLF5 protein can be degraded through ubiquitination-mediated pathways [34, 35], and we hypothesized that KLF5 loss with higher levels of stress in non-transformed epithelial cells might also be due to ubiquitin-mediated degradation Surprisingly, KLF5 ubiquitination decreased at later time points following UV exposure (Fig. 4B), coincident with increased expression of the deubiquitinases ATXN3L and BAP1 and decreased expression of the ubiquitinases FBW7, WWP1, and SMURF2 (Fig. 4C); notably, each of these regulate KLF5 degradation in other contexts [36–39]. ATXN3L, which was highly induced at the mRNA level following UV exposure, was also increased at the protein level (Fig. 4D). Thus, KLF5 is stabilized with UV stress, and KLF5 loss in response to higher levels of stress appears to be ubiquitin-independent, despite changes in the expression of several relevant ubiquitinases and deubiquitinases.

### KLF5 transactivates AKT to promote cell survival under stress

**Fig. 5:**
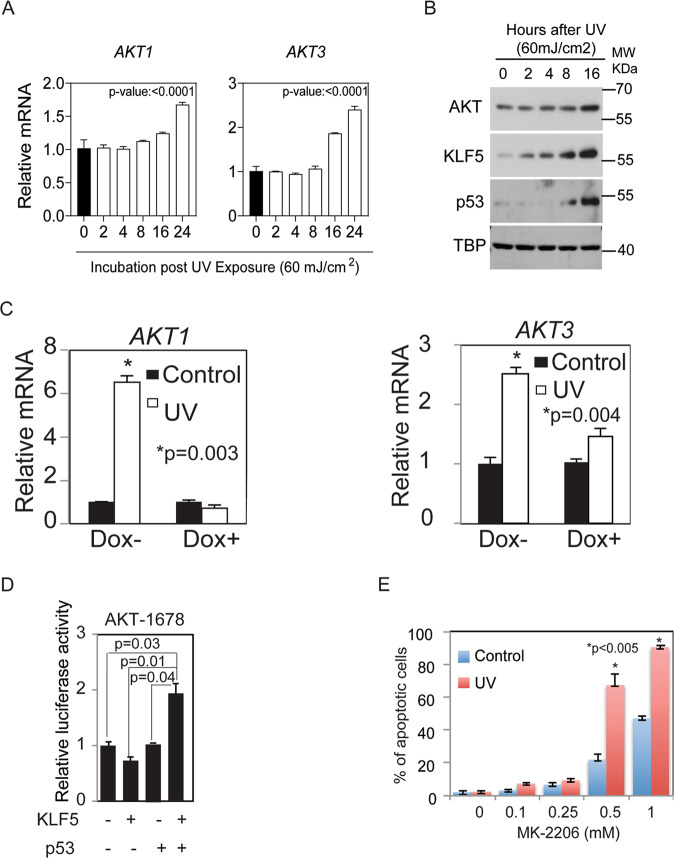
KLF5 and p53 cooperatively activate *AKT* in response to UV stress. **A** In primary human esophageal keratinocytes, *AKT1* and *AKT3* were induced starting at approximately 16 h after UV stress, as demonstrated by qPCR. **B** By Western blot, increases in KLF5 and p53 protein levels following UV stress preceded the induction of AKT. β-actin was a loading control. Western blots are representative of three independent biological experiments. **C** By qPCR, *AKT1* and *AKT3* were induced with UV stress, and this induction was blocked by *KLF5* knockdown. **D** Both KLF5 and p53 were required to transcriptionally activate *AKT*, as demonstrated with a luciferase reporter containing 1678 bp of the 5′ regulatory region of *AKT*. **E** Apoptosis increased significantly in primary human esophageal epithelial cells exposed to UV when AKT was inhibited with the AKT inhibitor MK-2206 for 8 h after UV irradiation (60 mj/cm^2^).

**Fig. 6:**
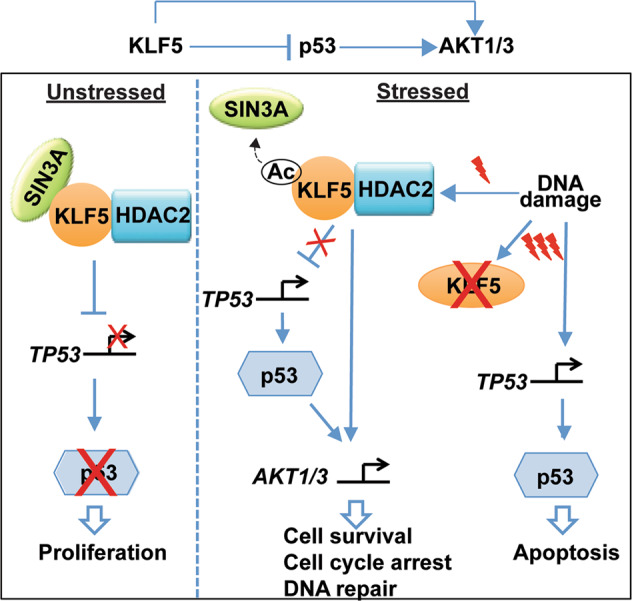
KLF5, p53, and AKT1/3 form an incoherent feed-forward loop that directs cell-fate decisions. In normal proliferating cells, KLF5 represses *TP53* via SIN3A and HDAC2. Under low levels of cellular stress, KLF5 is acetylated, leading to disruption of the KLF5-SIN3A-HDAC2 complex and de-repression of *TP53*. KLF5 and p53 together activate *AKT1* and *AKT3*, leading to cell cycle arrest, cell survival, and DNA repair. In contrast, higher levels of cellular stress led to KLF5 destruction through ubiquitin-independent pathways. In this case, while p53 is still de-repressed, KLF5 is no longer present, and thus *AKT1* and *AKT3* are no longer induced; as a result, cells undergo p53-mediated apoptosis.

The AKT pathway is a central regulator of cell survival in response to extracellular signals [40, 41]. A serine-threonine kinase with three highly homologous isoforms, AKT is activated through phosphorylation by PI3K signaling, but less is known about the mechanisms regulating *AKT* transcription, particularly in non-cancerous cells [40]. To determine whether AKT is a KLF5 target in primary epithelial during stress, we examined the regulation of AKT following UV stress. *AKT1* and *AKT3* we both upregulated by 16 h after UV exposure (Fig. 5A), and this induction of AKT was preceded by increases in both KLF5 and p53 (Fig. 6B). In addition, both *AKT1* and *AKT3* were induced by stress, and this induction was blocked by *KLF5* knockdown (Fig. 6C) indicating that KLF5 is required for *AKT* induction following UV stress. Moreover, transcriptional activation of *AKT1* required both KLF5 and p53 (Fig. 6D). To determine whether AKT was necessary for cell survival following stress, we blocked AKT activation with the allosteric AKT inhibitor MK-2206 in control and UV-irradiated epithelial cells. Consistent with a pro-survival function for AKT, apoptosis following UV irradiation increased with AKT inhibition in a dose-dependent manner (Fig. 6E). Similar effects were also observed with UV stress in other cell types, including primary skin and cervical cells (Fig. S8). Thus, KLF5 and p53 coordinately regulate *AKT1* and *AKT3* in human squamous epithelial cells, forming an incoherent feed-forward loop that dictates cell fate decisions in response to stress.

## Discussion

Understanding cell-fate decisions in normal cells and the role of p53 in these decisions requires insight into the mechanisms and consequences of p53 induction, including the network motifs that underlie p53 regulation and function. The feed-forward loop is a network motif that provides a means of tightly regulating gene expression and physiologic outputs in response to stimuli [42, 43]. By nature, feed-forward loops comprise two input transcription factors, one of which regulates the other and both of which jointly regulate a third factor. In a coherent feed-forward loop, the intermediate regulatory pathways have similar effects (i.e., activating or inhibiting). However, in other cases, the intermediate pathways have opposing roles; this type of network motif, known as an incoherent feed-forward loop, permits biphasic and potentially dose-dependent responses to a stimulus [44]. As such, an incoherent feed-forward loop constitutes an intriguing candidate mechanism for p53-dependent cell-fate decisions.

In the current study, we demonstrate that an incoherent feed-forward loop involving KLF5 and AKT1/3 regulates both p53 expression and function in normal human epithelial cells (Fig. 6). In normal unstressed epithelial cells, KLF5 complexes with SIN3A and HDAC2 to transcriptionally repress wild-type *TP53*, allowing cells to proliferate. In response to cellular stress, KLF5 is stabilized initially, but the KLF5-SIN3A-HDAC2 complex is disrupted, possibly through post-translational modifications; [24, 45] KLF5 then acts as a molecular switch for p53 function by transactivating *AKT1* and *AKT3*, which direct cells towards survival rather than apoptosis. However, increasing stress leads to the loss of KLF5, an inability to upregulate *AKT1* and *AKT3*, and preferential apoptosis. Taken together, these findings address a fundamental unanswered question in cellular stress and p53 biology; that is, how does p53 mediate cell fate decisions that dictate survival or death in response to stress [4, 11, 12]? While we have tested this model using primary squamous epithelial cells from the esophagus, skin, and cervix, sites of a large number of human cancers [46], it is not yet clear that this is applicable more broadly to other epithelial or other cell types. In addition, while the level of stress appears to be key in determining cell fate decisions, it is not certain if this is a threshold effect and if different types of stress can combine to drive cells toward apoptosis rather than survival. Moreover, the mechanisms of KLF5 loss by higher levels of stress are not yet clear, and delineating this pathway could have important therapeutic implications. Of note, small molecule inhibitors of KLF5 have been identified [47].

Cells normally respond to DNA damage by activating complex signaling networks that decide cell fate, promoting not only DNA repair and survival but also cell death [4, 48]. This decision between cell survival and death following DNA damage relies on proteins involved in DNA damage recognition, DNA repair, and damage tolerance, as well as others that activate apoptosis, necrosis, autophagy, and senescence. Induction of growth arrest rather than apoptosis is favored by the presence of pro-survival factors, and the selective expression of p53 target genes is central in the DNA damage response for survival and death [9, 48]. Certainly, some p53 target genes not studied here are likely to be important as well, and other factors could predispose cells directed towards growth arrest by KLF5, p53, and AKT to reenter the cell cycle, putting those cells at particular risk of sustained DNA damage and malignant transformation. Of note, a large number of p53 isoforms have been identified, and Δ113p53 transcriptionally activates *KLF5* and can contribute to the DNA damage response [49, 50]. Moreover, the pathways that are activated following stress may vary by the type of stress [51]. Finally, while KLF5 loss may promote apoptosis in both non-transformed and cancer cell lines, the outputs may differ, as KLF5 loss in HCT116 cells results in apoptosis through a p53-independent mechanism [52]. Thus, the network underlying cell fate decisions is unquestionably complex.

One surprising finding here is that, while KLF5 is stabilized initially following stress, associated with changes in expression of key regulators of ubiquitination and deubiquitination, ubiquitination of KLF5 decreases with increasing stress, even as KLF5 is lost. Interestingly, immunoprecipitation with KLF5 in the ubiquitination assay in Fig. 4C yields only a single band. Examination of a number of ubiquitinases and deubiquitinases known to interact with KLF5 [36–39] demonstrates that these deubiquitinases increase in expression while expression of the ubiquitinases decreases; this change in the balance between deubiquitination and ubiquitination of KLF5 may contribute to a lack of intense multimeric ubiquitinated KLF5. Also of note, KLF5 degradation may be independent of ubiquitination [53], and this decreased ubiquitination of KLF5 at higher levels of stress could be compensatory. Notably, lysine acetylation, as seen here for KLF5 during stress, can stabilize proteins by inhibiting ubiquitin-mediated protein degradation [54]. Interestingly, a specific human cancer-derived KLF5 is resistant to degradation [55], raising the possibility that, in response to higher levels of stress, this protein could direct cells that would normally undergo apoptosis to instead activate pro-survival pathways.

Differences certainly exist between the responses of normal human epithelial cells to stress and those of cancer cells, as well as the functions of KLF5, p53, and AKT in these responses. As such, our findings may be more relevant to cancer prevention than cancer treatment. For example, both p53 and the PI3K pathway are mutated in a large number of human cancers and may be functionally compromised in even more [8, 40]. Moreover, *KLF5* activating mutations can be seen in a diverse array of human cancers, in which *TP53* is also frequently mutated [32, 56, 57]. Nonetheless, some of the mechanisms of cell fate decisions in normal epithelial cells may be applicable to other contexts. Pharmacological targeting of p53 downregulatory pathways such as KLF5 may be relevant for cancers with wild-type p53. In addition, failure to activate AKT may lead to preferential apoptosis in response to stress, although the role of AKT in cell survival or death may vary by context [58]. Interestingly, in esophageal squamous cell cancer cells with mutant p53, constitutive KLF5 expression is sufficient to drive cells towards apoptosis; [21] thus, KLF5 may promote apoptosis in cancer cells through p53-independent mechanisms. Since we utilized only human squamous epithelial cells with UV and oxidative stress, it is possible that the regulatory mechanisms will differ in other cell types or in response to other cellular stressors. Thus, future studies will be needed to determine the broader impact of these pathways in cell-fate decisions following stress.

In sum, we identify here a novel regulatory mechanism for p53 and for epithelial cell responses to stress. Using non-transformed primary epithelial cells, we show that KLF5 is critical for *TP53* repression during normal cell proliferation and for determining the p53 output of growth arrest or apoptosis in response to cellular stress. Further delineating this pathway, including the upstream regulators of KLF5 and the downstream mediators of the response of normal cells to stress, has the potential to lead to new clinical diagnostic and therapeutic approaches.

## Supplementary information

Supplemental Figures

Table S1

Table S2

Table S3

Original Western blots

Reproducibility checklist

## Data Availability

All data generated or analyzed during this study are included in this published article (and its Supplementary information files).
