# Supplementary material for: KLF5 and p53 comprise an incoherent feed-forward loop directing cell-fate decisions following stress
Source: Cell Death Dis. 2023 May 2;14(5):299. doi: 10.1038/s41419-023-05731-1 (PMC10154356; doi:10.1038/s41419-023-05731-1)
Supplement: Supplementary file 1 — Supplemental Figures [file 41419_2023_5731_MOESM1_ESM.docx]

**Figure S1**. Locations and sequences of the primer pairs used for ChIP on the *p53* gene are shown (TS: transcriptional start site).

**Figure S2**. KLF5 knockdown promotes apoptosis and cell cycle arrest. (**A**) Flow cytometry with Annexin V staining demonstrated a progressive increase in apoptosis in primary human keratinocytes following KLF5 knockdown using a second shRNA, distinct from the one used in Figure 1C. (**B**) In primary human esophageal keratinocytes, *KLF5* knockdown resulted in progressive cell cycle arrest at G2/M. (**C-D**) While the p53 target *PUMA* (**C**) was increased at the transcriptional level by *KLF5* knockdown, other p53 transcriptional targets *BAD*, *14-3-3*, *GADD45A*, and *p21* (**D)** were not changed.

**Figure S3**. *TP53* knockdown decreases effects of *KLF5* knockdown on apoptosis and cell cycle arrest. (**A**) *TP53* was successfully knocked down using siRNA in human primary esophageal keratinocytes with *KLF5* knockdown by shRNA. Interestingly, KLF5 was also decreased with *TP53* knockdown suggesting the potential for reciprocal regulation. (**B**) *TP53* knockdown decreased the effects of *KLF5* knockdown on apoptosis. (**C**) Compared *KLF5* knockdown alone, *TP53* knockdown decreased the number of cells in G2/M.

**Figure S4**. Changes in p53 levels after KLF5 knockdown are not explained by alterations in (**A**) *MDM2* or *MDMX* expression or (**B**) *TP53* mRNA stability. Note that *TP53* mRNA stability actually decreased with *KLF5* knockdown, suggesting an attempt at compensation. TBP was used as an internal control.

**Figure S5**. KLF5 associates with SIN3A and HDAC2 including on *TP53*. (**A**) Mass spectrometry confirmed association of KLF5, SIN3A, and HDAC2. Peptide assignments to spectra were validated by the discriminant scores and false positive rates. (**B**) ChIP-ReChIP demonstrated binding of KLF5 with SIN3A and of KLF5 with HDAC2 to *TP53*. IgG served as a control.

**Figure S6**. KLF5 regulation with UV stress appears to be posttranscriptional, and KLF5 and p53 are also induced by oxidative stress, as with UV stress. (**A**) By qPCR, *KLF5* mRNA levels are initially decreased and then increased following UV stress, a different pattern than that seen for KLF5 protein levels after UV stress. (**B**) By Western blot, KLF5 and p53 increased when cells were treated with varying doses of hydrogen peroxide (H_2_O_2_) for 8 hours. (**C**) Similar to UV irradiation, hydrogen peroxide treatment disrupted the KLF5-SIN3A-HDAC2 repressive complex, as demonstrated by co-immunoprecipitation.

**Figure S7.** The KLF5 acetylation site at K369 is not required for *TP53* repression. (**A**) Full-length *KLF5* (KLF5-WT) and acetylation-deficient mutant *KLF5* (KLF5-K369R), which contains a lysine to arginine mutation at amino acid 369 of KLF5, were expressed in HEK293 cells, which lack endogenous KLF5. (**B**) KLF5-WT and KLF5-K369R, when co-expressed with SIN3A and HDAC2, repress the *TP53* promoter similarly. Note that KLF5, SIN3A, and HDAC2 expression are required for repression of the *TP53* promoter.

**Figure S8**. Effects of UV stress and the AKT inhibitor MK-2206 in other primary epithelial cells are similar to those seen with primary esophageal keratinocytes. In both primary skin (**A**, **C**, **E**) and cervical cells (**B**, **D**, **F**), *KLF5*, *p53*, and *AKT1* were induced by UV stress (**A-B**), apoptosis increased following UV irradiation as assessed by flow cytometry (**C-D**), and treatment with the AKT inhibitor MK-2206 significantly increased apoptosis resulting from UV stress (**E-F**). Cells were examined 8 hours after treatment with 60 mj/cm^2^ of UV.
