# Supplementary material for: KLF5 and p53 comprise an incoherent feed-forward loop directing cell-fate decisions following stress
Source: Cell Death Dis. 2023 May 2;14(5):299. doi: 10.1038/s41419-023-05731-1 (PMC10154356; doi:10.1038/s41419-023-05731-1)
Supplement: Supplementary file 2 — Table S1 [file 41419_2023_5731_MOESM2_ESM.docx]

| **Table S1. Sequences for short hairpin RNAs** | |
| --- | --- |
| *shNT forward* | CTA GCC CTA AGG TTA AGT CGC CCT CGT ACT AGT CGA GGG CGA CTT AAC CTT AGG TTT TTG |
| *shNT reverse* | AAT TCA AAA ACC TAA GGT TAA GTC GCC CTC GAC TAG TAC GAG GGC GAC TTA ACC TTA GGG |
| *shKLF5#1 forward* | CTA GCA CAG GGA ATA CAT TGT ATT AAT ACT AGT TTA ATA CAA TGT ATT CCC TGT TTT TTG |
| *shKLF5#1 reverse* | AAT TCA AAA AAC AGG GAA TAC ATT GTA TTA AAC TAG TAT TAA TAC AAT GTA TTC CCT GTG |
| *shKLF5#2 forward* | CTA GCG GCA ATT CAC AAT CCA AAT TTT ACT AGT AAA TTT GGA TTG TGA ATT GCC TTT TTG |
| *shKLF5#2 reverse* | AAT TCA AAA AGG CAA TTC ACA ATC CAA ATT TAC TAG TAA AAT TTG GAT TGT GAA TTG CCG |
