# Supplementary material for: KLF5 and p53 comprise an incoherent feed-forward loop directing cell-fate decisions following stress
Source: Cell Death Dis. 2023 May 2;14(5):299. doi: 10.1038/s41419-023-05731-1 (PMC10154356; doi:10.1038/s41419-023-05731-1)
Supplement: Supplementary file 3 — Table S2 [file 41419_2023_5731_MOESM3_ESM.docx]

| **Table S2. Sequences of siRNA** | |
| --- | --- |
| **Control siRNA** | rCrUrUrCrCrUrCrUrCrUrUrUrCrUrCrUrCrCrCrUrUrGrUGA |
|  | rUrCrArCrArArGrGrGrArGrArGrArArArGrArGrArGrGrArArGrGrAr |
| ***HDAC2* siRNA#1** | rGrArA rUrUrU rCrUrA rUrUrC rGrArG rCrArU rCrArG rArCA A |
|  | rUrUrG rUrCrU rGrArU rGrCrU rCrGrA rArUrA rGrArA rArUrU rCrUrC |
| ***HDAC2* siRNA#2** | rCrCrUrUrGrArArUrUrArCrUrArArArGrUrArUrCrArUrCAG |
|  | rCrUrGrArUrGrArUrArCrUrUrUrArGrUrArArUrUrCrArArGrGrArU |
| ***TP53* siRNA#1** | rArGrC rArUrC rUrUrA rUrCrC rGrArG rUrGrG rArArG rGrAA A |
|  | rUrUrU rCrCrU rUrCrC rArCrU rCrGrG rArUrA rArGrA rUrGrC rUrGrA |
| ***TP53* siRNA#2** | rGrArG rGrUrU rGrGrC rUrCrU rGrArC rUrGrU rArCrC rArCC A |
|  | rUrGrG rUrGrG rUrArC rArGrU rCrArG rArGrC rCrArA rCrCrU rCrArG |
