# Supplementary material for: KLF5 and p53 comprise an incoherent feed-forward loop directing cell-fate decisions following stress
Source: Cell Death Dis. 2023 May 2;14(5):299. doi: 10.1038/s41419-023-05731-1 (PMC10154356; doi:10.1038/s41419-023-05731-1)
Supplement: Supplementary file 4 — Table S3 [file 41419_2023_5731_MOESM4_ESM.docx]

| **Table S3. Primer sequences for quantitative real-time PCR** | | |
| --- | --- | --- |
| **Gene** | **Forward** | **Reverse** |
| *AKT1* | CAGTGCCAGCTGATGAAGAC | AAGGTGCGTTCGATGACAGT |
| *AKT3* | AAGGTGCGTTCGATGACAGT | AAGGTGCGTTCGATGACAGT |
| *ATXN3L* | TCCATTTTGGTCATCTGTCAA | AGTCCCACATCTCTCTCGTCA |
| *BAX* | CCCACAACAAAAGAGGTCCA | CCTGTCCATATTTGCAGGAGA |
| *BCL2* | GCCCTGTGGATGACTGAGTA | GGCCGTACAGTTCCACAAAG |
| *ESR2* | AGTCCCTGGTGTGAAGCAAG | ACGGTTCCCACTAACCTTCC |
| *FBW7* | ACGCCGAATTACATCTGTCC | TCTCTGGTCCACTCCAGCTC |
| *HAP1* | GAGGATGGGGTGCTGAGTC | CTGCCTTGGATTGGTCTGAT |
| *KLF5* | ACCCTGGTTGCACAAAAGTT | CAGCCTTCCCAGGTACACTT |
| *MDM2* | GGTGCTGTAACCACCTCACA | TTTTTGTGCACCAACAGACTTT |
| *p21* | CCAAGAGGAAGCCCTAATCC | CACAAACTGAGACTAAGGCAGAA |
| *p53* | AGGCCTTGGAACTCAAGGAT | TTATGGCGGGAGGTAGACTG |
| *PUMA* | ACGACCTCAACGCACAGTACGA | GTAAGGGCAGGAGTCCCATGATGA |
| *SMURF2* | CGCCTGACAGTACTCTGTGC | GATCCATCAACCACCACCTT |
| *TBP* | TGTACCGCAGCTGCAAAAT | GGATTATATTCAGCGTTTCG |
| *WWP1* | GCTTATGAACGCGGCTTTAG | CCGGGACACATTGATCTTTAC |
| *BAP1* | GAGGAGGTAGAGAAGAGGAAGAA | CTGAGCCAGCATGGAGATAAA |
