# Supplementary material for: KLF5 and p53 comprise an incoherent feed-forward loop directing cell-fate decisions following stress
Source: Cell Death Dis. 2023 May 2;14(5):299. doi: 10.1038/s41419-023-05731-1 (PMC10154356; doi:10.1038/s41419-023-05731-1)
Supplement: Supplementary file 5 — Original Western blots [file 41419_2023_5731_MOESM5_ESM.pptx]

## Slide 1
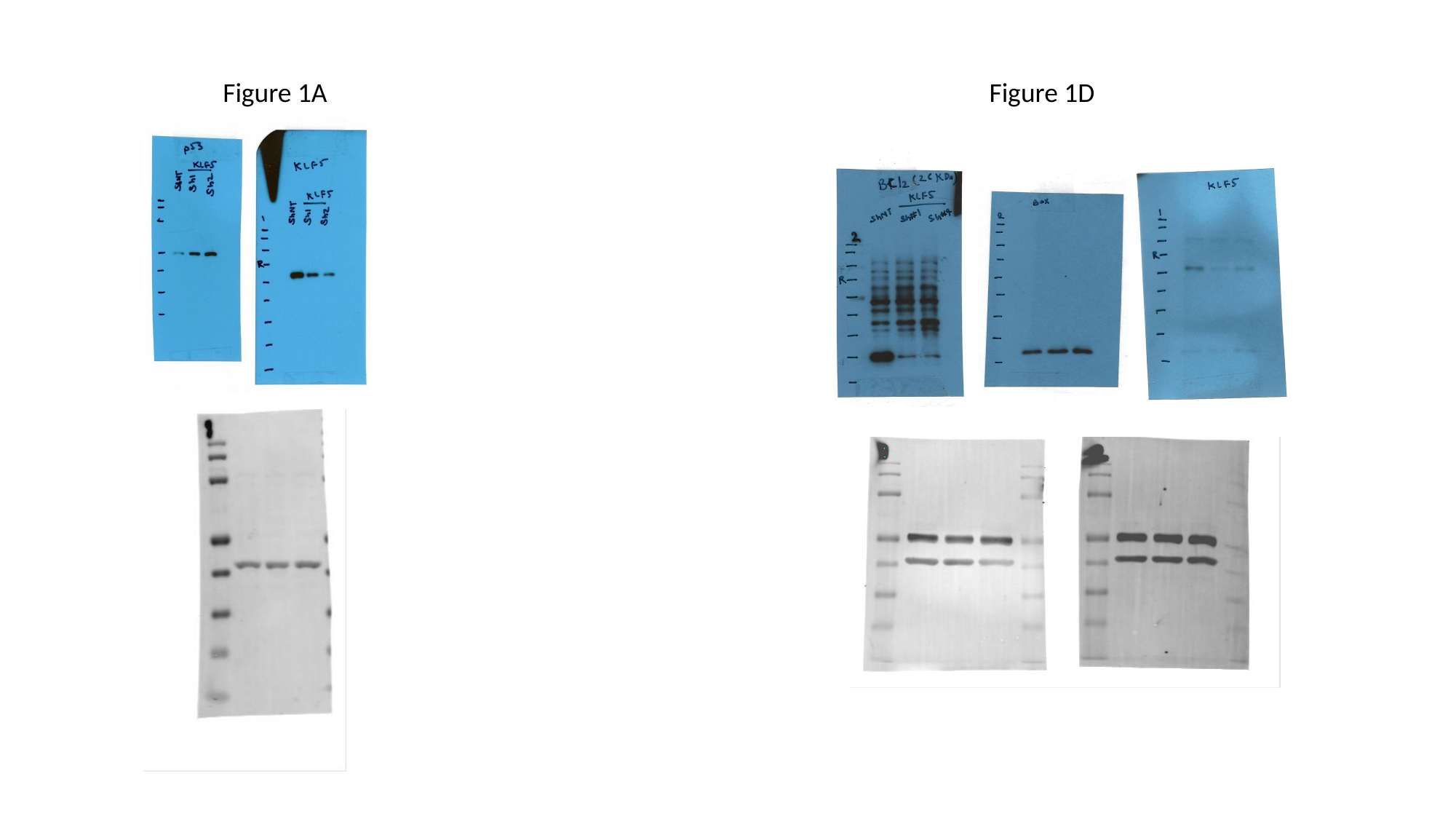

Figure 1A
Figure 1D

## Slide 2
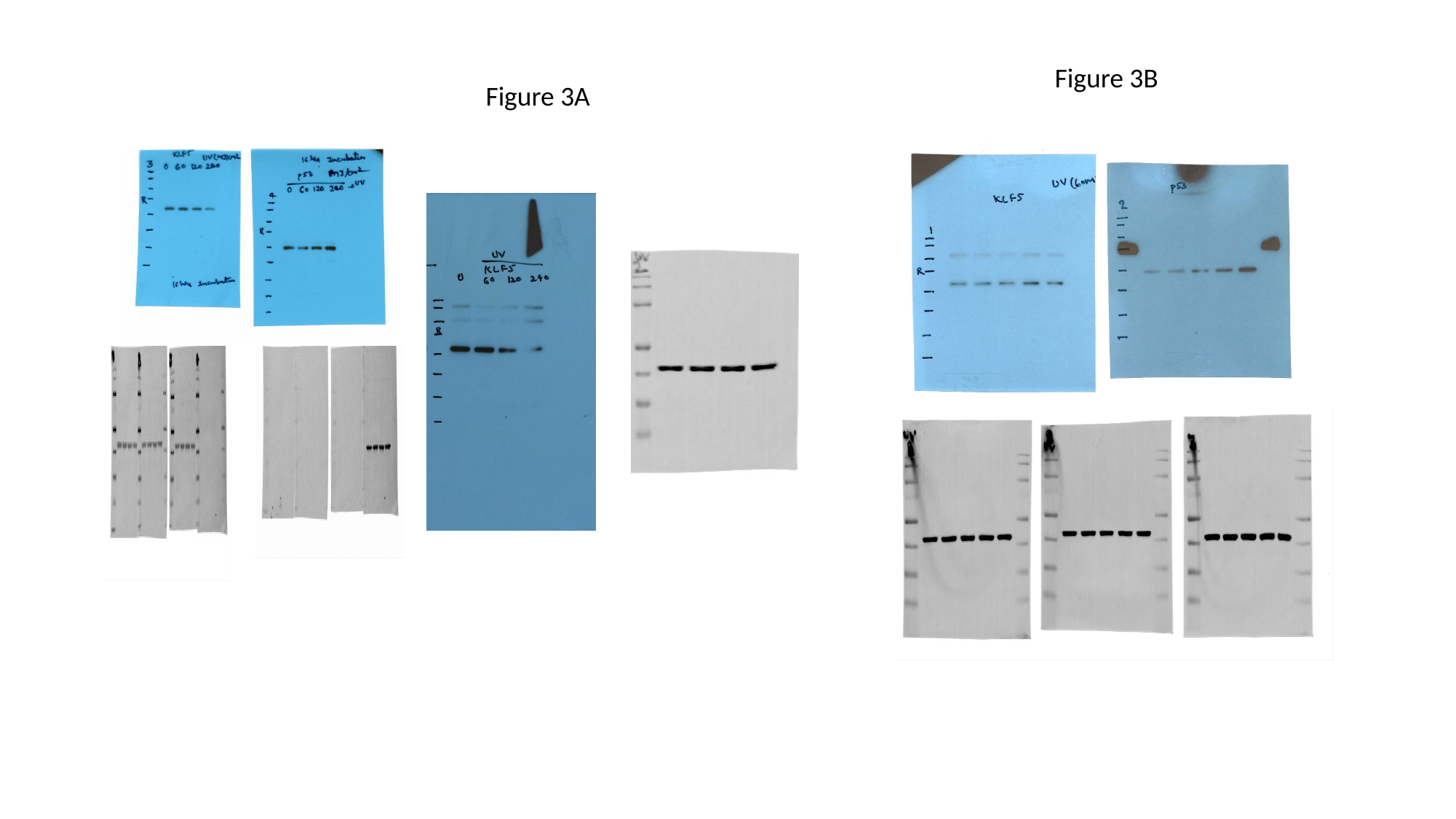

Figure 3B
Figure 3A

## Slide 3
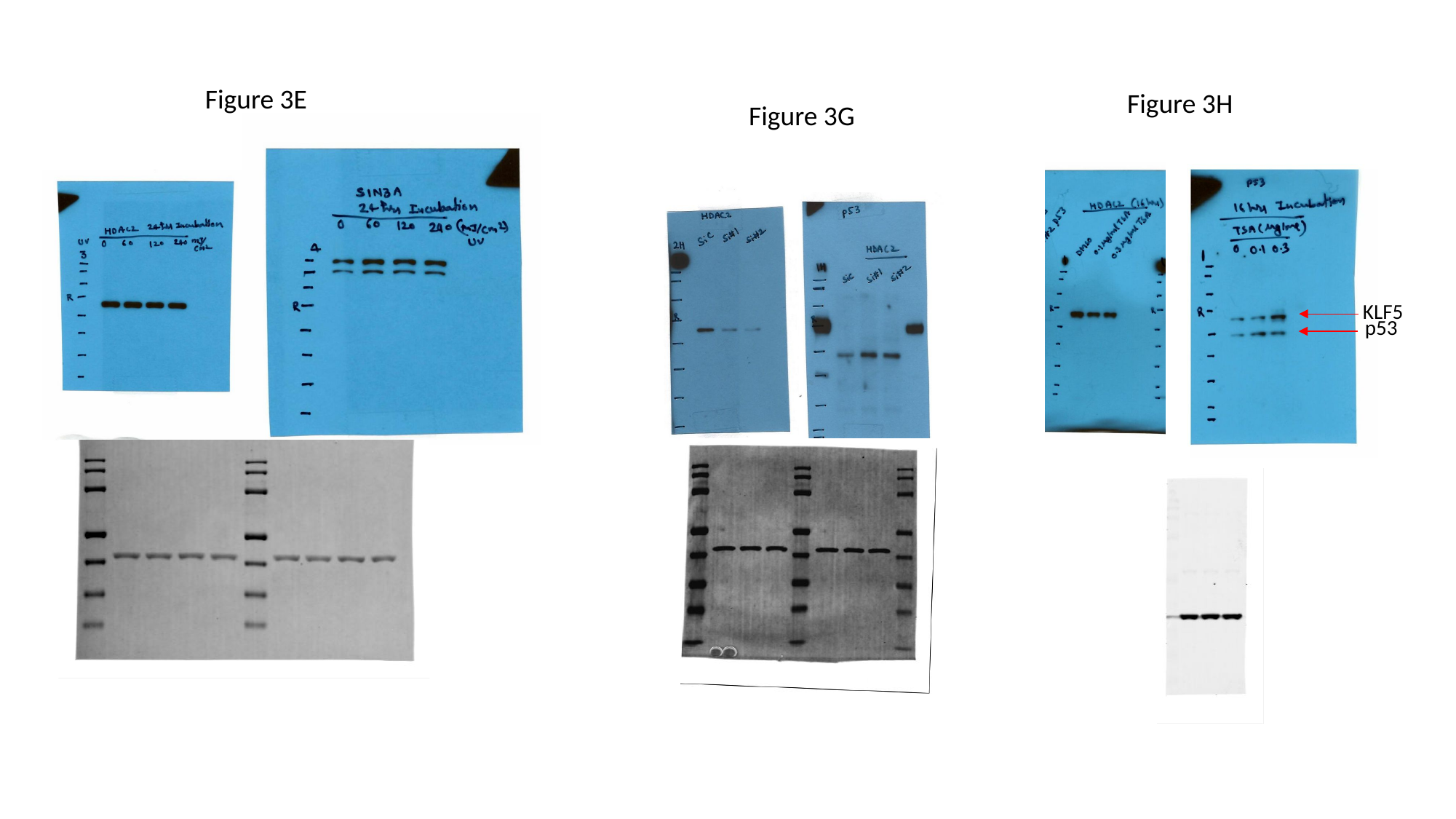

Figure 3E
Figure 3H
Figure 3G
KLF5
p53

## Slide 4
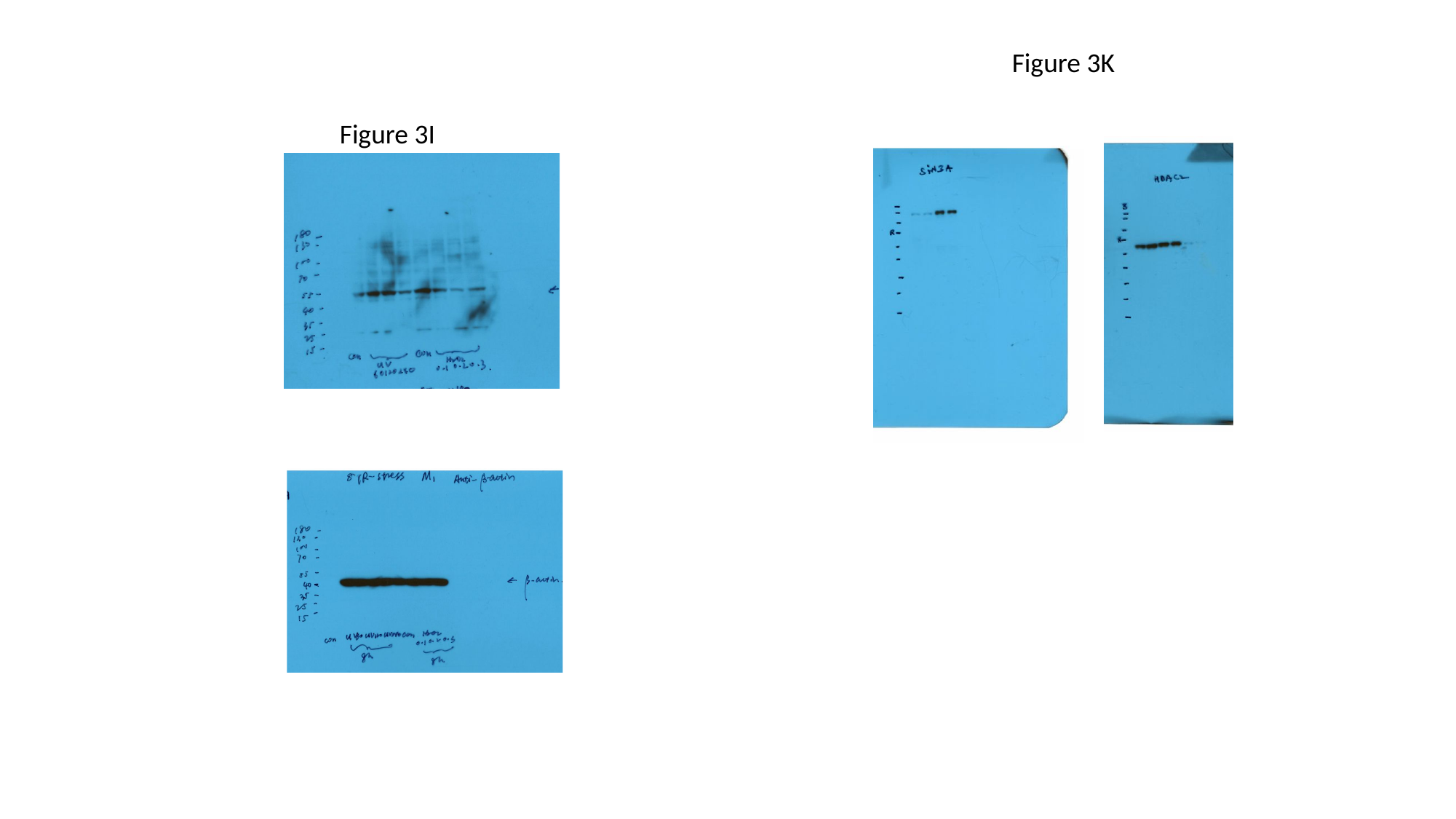

Figure 3K
Figure 3I

## Slide 5
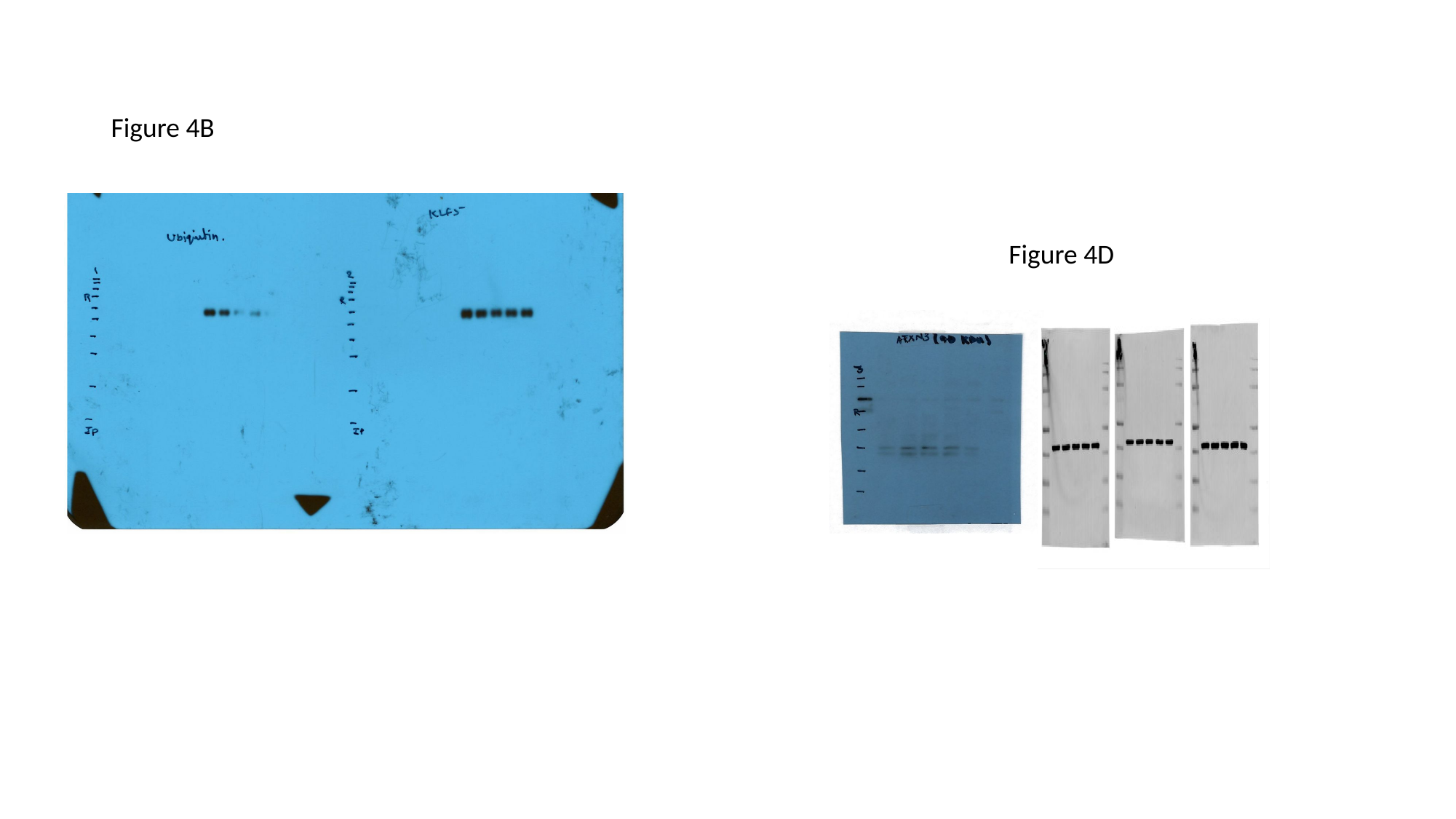

Figure 4B
Figure 4D

## Slide 6
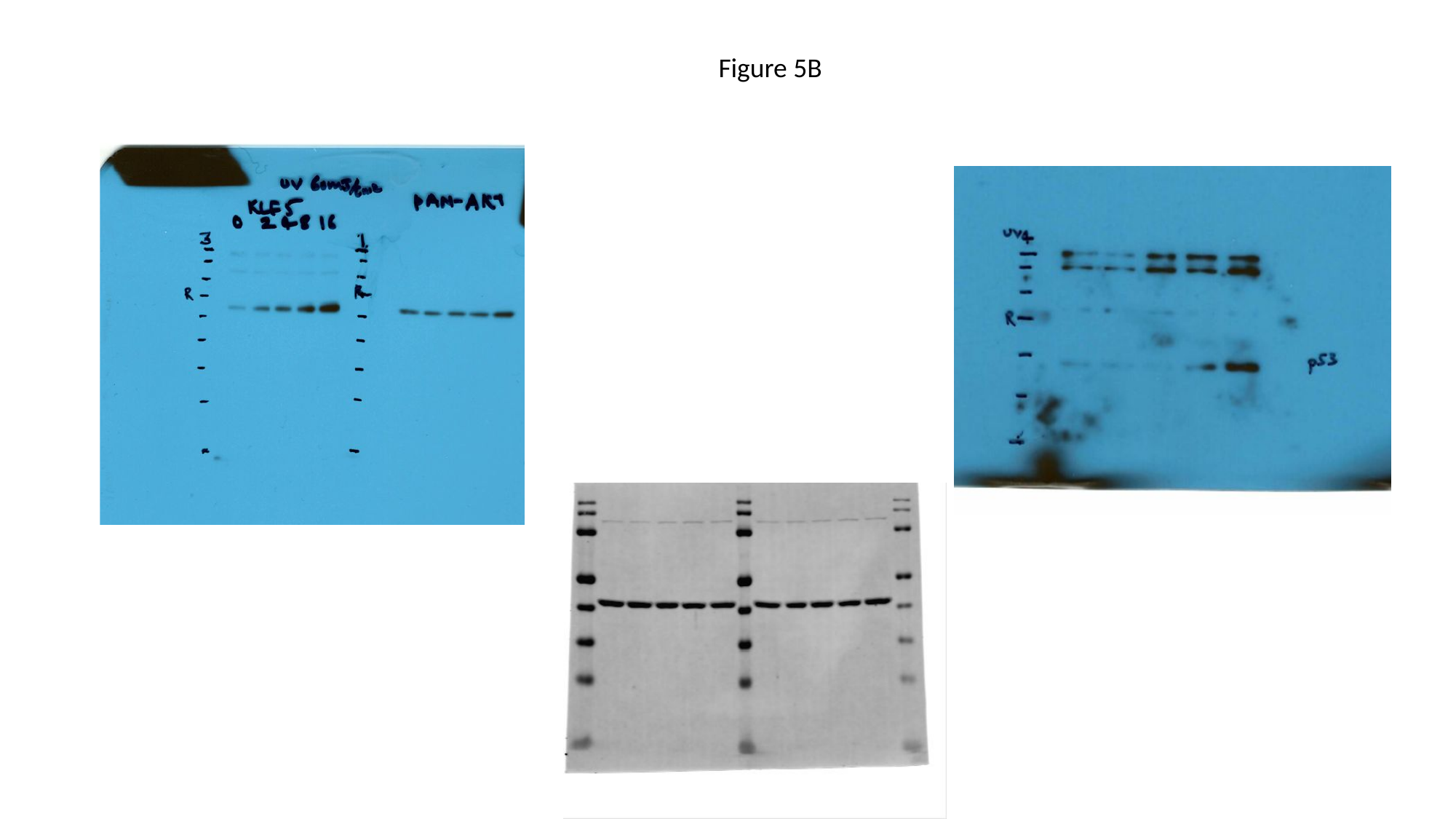

Figure 5B

## Slide 7
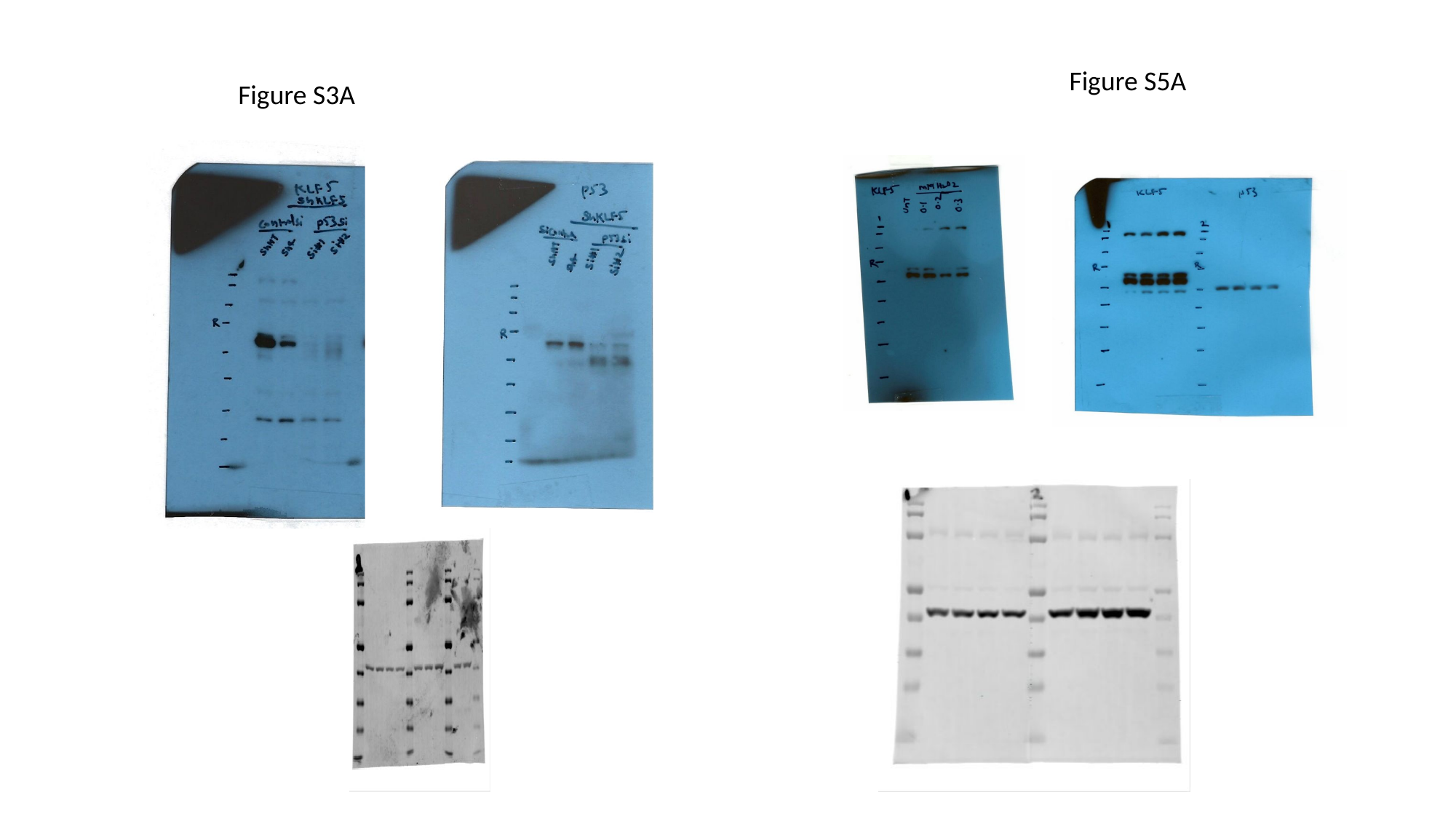

Figure S5A
Figure S3A
